# Abnormal Detection of Cash-Out Groups in IoT Based Payment

**DOI:** 10.3390/s21227507

**Published:** 2021-11-12

**Authors:** Hao Zhou, Ming Zhang, Lei Pang, Jian-Hua Li

**Affiliations:** 1 Institute of Cyber Science and Technology, Shanghai Jiao Tong University, Shanghai 200240, China; lijh888@sjtu.edu.cn; 2 Department of Risk Control, China UnionPay, No. 998 Jinxiu Road, CUP Tower, Shanghai 200135, China; zhangming@unionpay.com (M.Z.); panglei@unionpay.com (L.P.); 3 Shanghai Key Laboratory of Integrated Administration Technologies for Information Security, Shanghai 200240, China

**Keywords:** credit card transactions, cash-out group, Internet of Things (IoT), graph embedding, partial graph clustering, clustering, mobile devices, abnormal detection

## Abstract

With the rise of online/mobile transactions, the cost of cash-out has decreased and the cost of detection has increased. In the world of online/mobile payment in IoT, merchants and credit cards can be applied and approved online and used in the form of a QR code but not a physical card or Point of Sale equipment, making it easy for these systems to be controlled by a group of fraudsters. In mainland China, where the credit card transaction fee is, on average, lower than a retail loan rate, the credit card cash-out option is attractive for people for an investment or business operation, which, after investigation, can be considered unlawful if over a certain amount is used. Because cash-out will incur fees for the merchants, while bringing money to the credit cards’ owners, it is difficult to confirm, as nobody will declare or admit it. Furthermore, it is more difficult to detect cash-out groups than individuals, because cash-out groups are more hidden, which leads to bigger transaction amounts. We propose a new method for the detection of cash-out groups. First, the seed cards are mined and the seed cards’ diffusion is then performed through the local graph clustering algorithm (Approximate PageRank, APR). Second, a merchant association network in IoT is constructed based on the suspicious cards, using the graph embedding algorithm (Node2Vec). Third, we use the clustering algorithm (DBSCAN) to cluster the nodes in the Euclidean space, which divides the merchants into groups. Finally, we design a method to classify the severity of the groups to facilitate the following risk investigation. The proposed method covers 145 merchants from 195 known risky merchants in groups that acquire cash-out from four banks, which shows that this method can identify most (74.4%) cash-out groups. In addition, the proposed method identifies a further 178 cash-out merchants in the group within the same four acquirers, resulting in a total of 30,586 merchants. The results and framework are already adopted and absorbed into the design for a cash-out group detection system in IoT by the Chinese payment processor.

## 1. Introduction

Consumption using a credit card has become more and more popular, bringing convenience, safety and speed to people’s daily lives. However, this also fosters the abnormal behavior of cashing out funds from credit cards. Cash-out with credit cards in this paper refers to a situation in which credit cardholders obtain cash through transactions either face-to-face or online, mostly using mobile devices instead of an ATM or counter. Specifically, the merchant receives the funds after transaction settlement by the acquirer and pays the funds back to the credit cardholder, charging the handling fee. In mainland China, the credit card transaction fee is, on average, lower than the retail loan rate, and it is easier to have a credit card approved than a small retail loan. Thus, credit card cash-out is attractive for investments or business operations, which are considered unlawful if exceeding a certain amount.

With the development of Internet of Things (IoT) in different areas, such as sensor networks [1] and radiation constrained scheduling [2,3], etc., more and more scholars have conducted research on new technologies in IoT combined with AI (artificial intelligence). Blockchain-based risk blacklist sharing is under research, which could solve the problem of finding a balance between data sharing and data protection. A software-defined blockchain architecture is proposed to realize the dynamic configurations for blockchains in IoT [4]. Some scholars research data stream mining [5] and security and privacy of edge computing [6,7,8]. Others propose a novel Wirelessly Powered Edge intelliGence (WPEG) framework, which aims to achieve stable, robust, and sustainable edge intelligence by energy harvesting (EH) methods [9]. It is recommended to use a fast payment based on credit to enhance the efficiency of computing resources trading [10]. A scheme which takes advantage of the merits of Android Pay and a refined certificateless signature cryptosystem to simultaneously deliver transaction security and achieve payment efficiency in practice under Internet of Things (IoT)-based network architectures is proposed [11]. In IoT-based payment, payment devices and online merchants have closer relations than in physical payment.

Generally, financial institutions use expert experience to set risk rules, to analyze transaction data, and to filter suspicious credit cards or merchants in order to identify fraud and abnormal behavior. This traditional method has shortcomings in terms of detecting cash-out groups, as follows. (1) Expert experience can be easily explored by the cash-out group chain and thus can be avoided in a targeted manner. (2) Expert rules are effective at identifying cash-out cards or merchants with unchanged characteristics, but are not effective at identifying out cash-out cards or merchants in groups without obvious unchanged characteristics. (3) Expert experience often lags behind the fast-changing cash-out modes, which are adjusted manually rather than automatically.

### 1.1. Related Work

Since the 1980s, data mining technology has developed and more technicians have applied this technology into the field of financial risk control. In credit card transaction fraud detection, methods like SVM (support vector machines), neural network, and RF (random forest) have emerged [12,13,14,15,16,17]. Although these methods have relatively good results in terms of targeting suspicious credit card transactions, they cannot solve the problem of abnormal detection of groups, rather than individuals. For cash-out groups, it is difficult to confirm suspicious activity due to the cardholder and the conspired merchant not actively declaring the behavior because it results profit for each side and no loss. Therefore, supervised learning algorithms for fraudulent credit card cash-out detection [18] requiring precise and overall labels of fraudulent transactions do not work well in cash-out group detection.

At present, there are two main methods for detecting credit card fraud using machine learning algorithms in the industry: supervised learning and unsupervised learning. The former trains the model based on fraud samples and normal samples, thus calling for sufficient and updated positive and negative samples. The latter classifies abnormal transactions into different categories of fraud using a clustering algorithm to put transactions into groups. Both supervised learning and unsupervised learning could predict the probability of credit card fraud. Sometimes, they are mixed in use.

Supervised learning: As the application of associated network technology has become more widespread, it can effectively express the association between nodes in a graph. More and more scholars and technicians have begun to try to detect credit card fraud based on the natural bipartite graph formed by credit card transactions in merchants, and use graph-based data mining algorithms. Some researchers [19] decomposed the credit card-to-merchant bipartite graph into multiple subgraphs, and adopted a divide-and-conquer strategy for fraud detection. Others [20] used the cardholder-merchant bipartite graph to estimate the probability of each user being involved in credit card fraud or counterfeiting in the framework of the Markov Random Field (MRF). Some scholars [21] added device information to the bipartite graph, considering two meta-paths, including cardholder–cardholder and cardholder–merchant–cardholder, and designed a neural network with an attention mechanism for learning the embedded representation of each node and predicting the probability of cardholder fraud. The above methods are essentially supervised learning [22,23,24,25,26], and predict the fraud probability of nodes (cardholders or merchants) through node attributes and related information between nodes.

Unsupervised learning: Without a confirmation label, it is a typical unsupervised learning problem [27,28,29,30,31]. Some scholars [32] propose a model to analyze abnormal patterns of transactions over the payment network, discovering four types, which are: high-risk merchants, marketing promotion fraud card, cash redistribution network and group fraud. Others [33] build a transaction graph network based on financial transaction data, and establish a topological graph feature extraction framework and abnormal detection model. Group detection of credit card cash-out is essentially an unsupervised problem. The cash-out merchant group is more critical and hidden than the cash-out card group.

We creatively propose a weakly supervised learning method based on the association network technology combining supervised learning and unsupervised learning. First, the seed cards are mined through rule-based methods, and the seed cards’ diffusion is then performed through the partial graph clustering algorithm (Approximate PageRank, APR), which produces a batch of suspicious cards. Second, a merchant association network is constructed based on the suspicious cards. The graph embedding algorithm (Node2Vec) is used to represent and learn the merchant as nodes, in order to map the topological association between the merchants into the vector space. Third, we use the clustering algorithm (DBSCAN) to cluster the nodes in the Euclidean space, which divides the merchants into groups. Finally, we design a method to classify the severity of the groups to facilitate the following risk investigation.

### 1.2. Motivation

We target three tasks, as follows.

- To identify more cash-out groups who are more hidden than individuals, and often cross multiple acquirers who provide the service to merchants.
- To propose a method for classifying groups into different priorities to facilitate further investigation, providing explainable features.
- To support large scale data processing in order to enable implementation as a real-time system.

### 1.3. Contributions

The main contributions of the paper are summarized as follows.

- In order to mitigate the insufficiency and incompleteness of cash-out risk rules, we construct an association network between cards using a partial graph clustering algorithm to spread the seed cards into a set of suspicious cards.
- We construct an association network between merchants through credit card transaction data, using a graph embedding learning algorithm (Node2Vec) and clustering algorithm (DBSCAN) to identify cash-out merchant groups.
- The merchant and credit card network reaches more than one million nodes representing merchants and more than six million edges showing the similarity of nodes, covering hundreds of millions level transaction information.
- We design a group severity rating system from the perspective of engineering application, taking into account group aggregation and group severity, and verifying the rationality of the rating system through a known dataset.

The rest of the paper is organized as follows. In Section 2, we present the system model. Section 3 presents the results. Discussions are presented in Section 4, and Section 5 concludes the paper.

## 2. System Model

**Figure 1:**
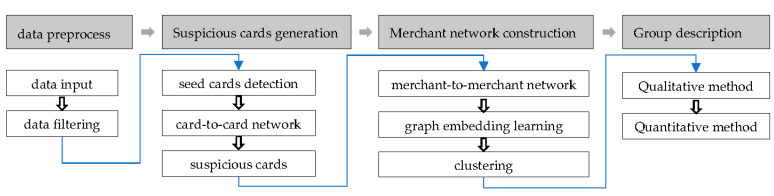
Flow chart of model setup.

We consider that there are a significant amount of data and merchant-to-merchant networks are more difficult to detect. Therefore, we develop models covering the four major parts in Figure 1. The model is different from the existing single algorithm-based method, like supervised learning or unsupervised learning. The model firstly proposes the combination of expert rule, graph embedding and unsupervised learning.

### 2.1. Algorithm and Terms

Several algorithms are used in this paper, including Approximate PageRank (APR), Node2Vec and DBSCAN. Table 1 provides a description of the terms used in this paper:

Approximate PageRank (APR) [34,35] algorithm is a personalized ranking algorithm based on the random walk model, but with made some improvements to the original PageRank algorithm. Different from the original PageRank algorithm—which calculates ranking as a whole—APR performs a random walk on the nodes of interest and a local personalized ranking. Suppose $p_{1},\ldots {,p}_{N}$ are nodes, ${M(p}_{i})$ is the set of nodes relevant with node *P_i_*, ${L(p}_{j})$ is the number supremum of the nodes relevant with node *P_j_*.

When *t* = 0, the initial probability distribution is ∀1≤i≤N, and then

(1) $${PR(P}_{i};0)=\frac{1}{N}$$

With the time goes, the *PR* value in each step can be written as:

(2) $${PR(P}_{i};t+1)=\frac{1-d}{N}+d\sum_{p_{j}\in {M(p}_{i})}\frac{{PR(p}_{j},t)}{{L(p}_{j})}$$

where *d* is the transition probability coefficient, *d*∈(0,1) and is a constant related with the number of edges between nodes.

The APR algorithm starts from a given node or a set of seed nodes, walking in a first-order random walk in the network, and continuously expanding outwards for eligible communities for directional clustering, without consideration of the size of the whole graph. Generally speaking, the higher the *PR* value, the higher the similarity between the representative node and the seed node.

**Figure 2:**
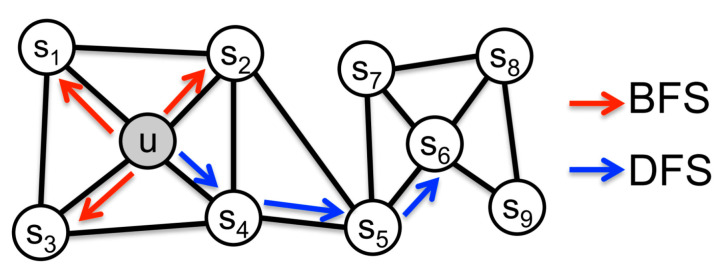
BFS and DFS in Node2Vec.

Node2Vec [36] is used to learn the continuous feature expression of network nodes, mapping to low-dimensional feature space and preserving the neighborhood of nodes in the network to the greatest extent. Node2vec proposes a biased random walk, using two graph walk methods which are breadth first search (BFS) and depth first search (DFS) in Figure 2. BFS tends to wander near the immediate neighbor nodes, which can reflect the microscopic characteristics of a node’s neighbors; DFS tends to wander farther, which can reflect the macroscopic characteristics of a node’s neighbors. By citing two hyper parameters *p* and *q* to balance BFS and DFS, the random walk is guided in Equation (3), where *p* represents the possibility of repeated wandering, and *q* represents the possibility of visiting other nodes that are farther away from the node.

(3) $$P(c_{i}=x|c_{i-1}=v)=\{\begin{array}{cc} \frac{\alpha_{pq}\left(t,x\right)\times w_{\mathrm{vx}}}{Z} & \mathrm{if}\;(v,x)\in E\\ 0 & \mathrm{otherwise} \end{array}$$

(4) $$\alpha_{\mathrm{pq}}(t,x)=\{\begin{array}{cc} \frac{1}{p} & \mathrm{if}\;d_{t,x}=0\\ 1 & \mathrm{if}\;d_{t,x}=1\\ \frac{1}{q} & \mathrm{if}\;d_{t,x}=2 \end{array}$$

where *d_t,x_* represents the shortest distance from node *t* to node *x*, *w* is the edge weight.

Density-Based Spatial Clustering of Applications with Noise (DBSCAN) [37] is a density-based spatial clustering algorithm which defines clusters as the largest collection of points connected by density. It can divide regions with sufficient density into clusters, and can identify arbitrary shaped clusters in noisy spatial datasets. The basic idea is that for each object in a class, the number of objects contained in the area of a given radius *r* cannot be less than a given minimum number of *min_points*. The algorithm steps are as follows:

Step1.

Choose an unvisited point to start, and find all nearby points within *r*.

Step2.

If the number of nearby points is greater than or equal to *min_points*, the current point is the core point, then, recursively, process all of the unmarked points in the cluster in the same way, and identify all the data whose density can be reached from the point, forming a cluster.

Step3.

If the point is a noise point, temporarily mark it as a noise point and select another data point.

Step4.

The cluster is fully expanded—that is, all points in the cluster have been visited—use the same algorithm to deal with unvisited points.

Repeat steps 2, 3, and 4 until all points are processed.

### 2.2. Data Preprocessing

Considering computation complexity, this step is intended to filter irrelevant data with cash-out behavior from the original credit card transaction data, such as transactions with very low amount or those which happened overseas, as these will generate a certain noise for the identification of credit card cash-out groups.

Data filtering is used to improve data quality and to ensure the reliability of cash-out group mining. After data preprocessing, approximately 10% to 15% of irrelevant transactions will be excluded.

### 2.3. Suspicious Cards’ Generation

After data filtering, the cards and merchants are still both big scale with a minor portion as cash-out groups. Thus, it is very difficult to identify cash groups from the data. We try to identify suspicious cash-out cards with abnormal characteristics in order to narrow the scope of analysis. Following this, only those merchants with suspicious cards who are considered to be relevant would be analyzed prior, which can not only make full use of the resources, but can also reduce noise interference to a certain extent.

#### 2.3.1. Seed Cards Detection

Cash-out cards often have the following characteristics: (1) frequent transactions in a short time period; (2) monthly periodic transactions; (3) consecutive transactions with a similar or equal amount; (4) transactions within an abnormal time; and (5) transactions in and out with a similar amount, alternatively. Through the above characteristics, we locate cash-out cards as seed cards by expert rule from the massive transaction data.

#### 2.3.2. Card-to-Card Network

Generally, cash-out cards would swipe back and forth among the multiple merchants of which the cash-out group is composed, from a high efficiency perspective. Conversely, cash-out merchants would be more hidden by mixing cash-out transactions and normal transactions. However, some cards and seed cards are owned by the same merchants, meaning that similar transactions would happen in the merchants. We call these cards suspicious cards which are hidden and cannot easily be identified through expert rule.

**Figure 3:**
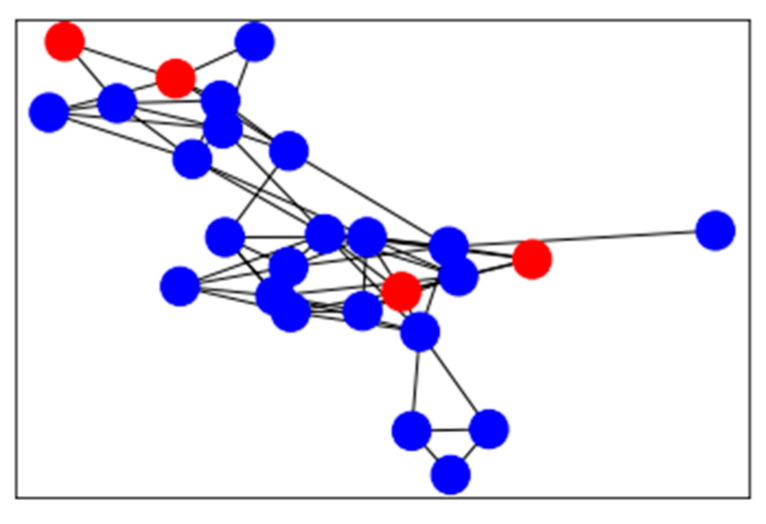
Card-to-card association network graph.

Considering the huge scale of cards, it is unrealistic to analyze suspicious cards through seed cards based on the shared merchants one by one. Local graph clustering aims to explore the local area of the graph, starting from a given seed node and expanding outward, looking for the community in which the seed node is located for directional clustering, without considering the size of the whole graph. Based on this, the association between seed cards and suspicious cards is created according to timely sequential transactions at the same cash-out merchant, and thus a card-to-card association network is constructed, as shown in Figure 3. The number of timely adjacent transactions at the same merchant is used as the edge weight, while the card is used as the node within the card-to-card association network. Since the time complexity of local graph clustering only depends on the seed cards instead of the totality of cards for which there are data, it is possible and efficient to identify suspicious cards. In Figure 3, the red nodes are seed cards and the blue cards are suspicious cards which have been found.

By comparing several common local graph clustering algorithms, such as approximate PageRank (APR), HOSPLOC and MAPPR, we finally chose APR Algorithm 1, because it is more efficiently than others, in terms of both space and time.
**Algorithm** **1.** APRApproximatePR (*s,*
*α, ε*): 1. Let *p* = $\vec{0}$, and *r* = *s*
2. **while** *r*(*u*) ≥ *εd*(*u*) for some vertex *u*: (a) Pick any vertex *u* where *r*(*u*) ≥ *εd*(*u*) (b) push(*u*): Let *p*’ = *p* and *r’* = *r*, except for these changes: *p*’(*u*) = *p*(*u*) + *α r(u)*
*r’* (*u*) = (1-*α*)*r*(*u*)/2 For each vertex *v* such that (*u, v*) ∈ *E:*
*r’* (*v*) = *r*(*v*) + (1-*α*)*r*(*u*)/(2*d*(*u*)) Update *r* = *r’,*
*p*
*=*
*p*’ 3. **return**
*p and r*

### 2.4. Merchant Network

#### 2.4.1. Merchant-to-Merchant Network

The suspicious cash-out cards narrow the scope of cash-out merchant group mining into its associated merchants, and, as such, building the relationship between merchants becomes another key. Under normal circumstances, there are many shared cash-out credit cards among the cash-out merchants in a group. Therefore, based on the seed and suspicious cards, the relationship between the merchants can be constructed, and the correlation degree is considered using the following three methods:

- Method 1: Using the number of shared seed cards and suspicious cards among merchants to measure the closeness of merchants is simple and intuitive, but different cards may have very different transaction amounts. Thus, it cannot reflect the difference in transaction amount.
- Method 2: Using the total transaction amount of shared seed and suspicious cards among merchants improves the flaw caused by Method 1. However, there are differences in the scale of merchants. Large retail merchants have a large daily transaction amount while small convenience shops have a smaller amount. The total transaction amount may appear insignificant for large merchants, and the impact in terms of merchant scale is to be considered.
- Method 3: Combined with the strength of Method 1 and Method 2, the number of shared seed cards and suspicious cards and the ratio of amount with these cards to the total amount with the merchants are considered.

**Figure 4:**
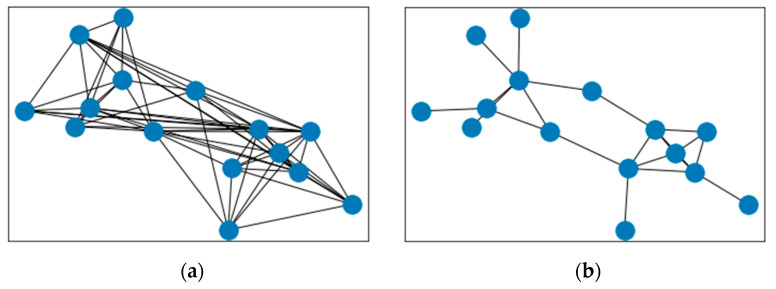
Merchant-to-merchant network4, (**a**) merchant network by method 14; (**b**) merchant network by method 3.

After comparison, Method 3 is the best choice, as shown in Figure 4. In Figure 4, the nodes represent merchants with shared seed and suspicious cards. Figure 4b has filtered weakly related connections in the original merchant network in Figure 4a.

#### 2.4.2. Graph Embedding Learning and Clustering

To detect groups in a network through network topology, we chose the methods of graph embedding learning and clustering together.

As a graph embedding learning algorithm, Node2vec is used to select the next wandering node in the merchant network with the edge weight as the probability, carrying out the embedded representation learning of the merchant network nodes, and mapping the network topology relationship to the vector space. The merchants of the same group are closely connected and related, and have similar expressions.

**Figure 5:**
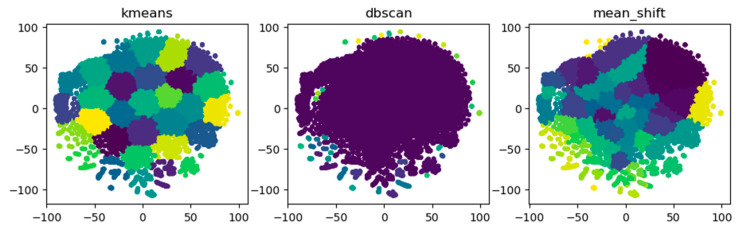
Group detection of three clustering algorithms.

As clustering algorithm, DBSCAN is chosen which can effectively deal with noise while K-means needs to manually specify the number of cluster centers and Mean-Shift [38] depends on the choice of bandwidth. Table 2 compares the three clustering algorithms. The merchants in the Euclidean space indicate that the nodes in the vector space are clustered using traditional clustering algorithms, and each node is divided into different clusters. Figure 5 shows that DBSCAN can better capture the abnormal groups on the edge, while the other two algorithms could not differentiate abnormal merchants on the edge from normal merchants in the middle with the same color.

### 2.5. Group Description

In order to describe the group in more detail, we obtain basic information in different aspects, such as the merchants in the group, cards in the group and the network structure, in order to construct a portrait of the cash-out group.

#### 2.5.1. Qualitative Description

Usually, classification of cash-out groups is necessary for understanding the groups.

- From the transaction amount scale perspective, the big, medium or small group could be differentiated.
- From the characteristics of the shared cash-out cards (mostly seed cards), the big-amount, QR-code based transaction, credit card balance for circular use may be the typical types.
- From the type of the acquirer who provides the acquiring service to the merchants, merchants acquiring by bank and merchants acquiring by non-bank are the two different classes.
- For some groups, the above characteristics are combined, and these can be referred to as a combined type.

#### 2.5.2. Quantitative Description

To be more clear, quantitative methods are used to describe and rank the cash-out groups. We assume two factors—closeness in group and scale in transaction—to reflect the composite rank of the groups after mining.

- *Closeness.* Based on the network topology of the merchant nodes, it is possible to calculate the average clustering coefficient *C* and the average amount of sharing in the shared cash-out cards *NumS*, etc. We then normalize these to the linear weight, measuring the closeness of the network connection.
  (5) $$\mathrm{Closeness}=C\times {\mathrm{Weight}}_{c}+\mathrm{NumS}\times {\mathrm{Weight}}_{\mathrm{nums}}$$
  where *weight_c_* and *weight_num_* are the weight value, with the default value is 60% and 40% separately.
- *Scale.* We use the total transaction amount *A_total_,* shared cards’ transaction amount *A_shared_*, and the number of shared cards *NumC* as three parameters to calculate the scale of groups by normalization.
  (6) $$\mathrm{Scale}=A_{total}\times {\mathrm{Weight}}_{a}+A_{shared}\times {\mathrm{Weight}}_{\mathrm{as}}+\mathrm{NumC}\times {\mathrm{Weight}}_{\mathrm{nc}}$$
  where *weight_a_*, *weight_as_* and *weight_nc_* are the weight value, with the default value is 40%, 40% and 20% separately.

*Rank calculation.* After the calculation of closeness and scale, we divide the results into three categories (A, B, C) separately according to a certain ratio, such as normal distribution, from the high to the low. The rank is then produced by combining the categories of the two factors, such as AA, AC, etc. AA refers to the group which needs to be investigated as the highest priority, while CC means the lowest priority.

## 3. Results

### 3.1. Dataset and Evaluation Method

We use 6-month real transactions including IoT devices provided by a worldwide payment processor headquartered in China. Each transaction includes the entire message (shown in Table 3) after data protection of key data, like PAN (Primary Account Number), Merchant code, and Mobile device information, etc. After data preprocessing, 3.28 billion transactions are entered into the model. The graph has one million nodes and more than six million edges in the experimental environment. It is clear that 195 known risky cash-out merchants in a group are in the dataset, because it is after investigation by police. In order to verify the effectiveness of this model, the main work is as follows.

- We try to verify the effectiveness of the local graph clustering by comparing the results with the suspicious cards and without the suspicious cards.
- We introduce the cash-out merchant precision and the case hit rate as the evaluation index. Precision refers to the percentage of the accurate cash-out merchants that the model recognizes and that can be confirmed. The case hit rate refers to the percentage of the number of cash-out merchants that the model covers within the known 195 merchants.

### 3.2. Platform Infrastructure

As we need to provide support which is large-scale and efficient, real-time updated computing, storage and query, distributed system is used based on Hadoop, HIVE, HDFS, Hbase. ArangoDB as graph database is used. We use six servers (16 Cpu, 64G storage), eleven servers (8 Cpu, 32G storage) and three servers (4 Cpu, 8G storage). The data can be updated on a daily basis. Taking ROI (return over investment) into consideration, we update the data once a month because the cash-out group remains almost the same in a month.

### 3.3. Experimental Results

We sets up a series of expert rules from a variety of cash-out modes, such as large amount mode, periodic mode, abnormal mode, balance for circular use mode (balance mode in short), and consecutive transactions with similar or equal amount balance mode (equal amount mode in short). The meaning of the above five modes are as follows.

- Large amount mode: the credit card transaction amount is bigger than the average amount normally used in the merchant.
- Periodic mode: the credit card transactions happen monthly, especially partly or wholly close to a fixed date or a set of fixed dates, which is perhaps the latest repayment date.
- Abnormal mode: any abnormal transaction in time or in frequency, etc.
- Balance for circular use mode: a certain skill making full use of the small balance between money out and money in.
- Equal amount balance mode: the single or total transaction amount is same or similar.

The seed cards are selected from the above expert rules (Table 4).

#### 3.3.1. The Effectiveness of Suspicious Cards

**Figure 6:**
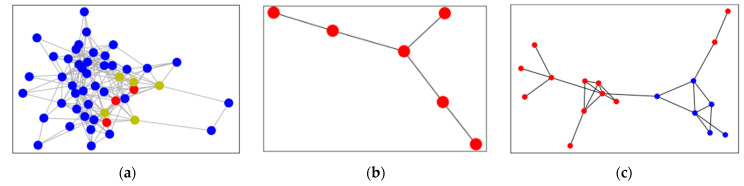
Analysis on card groups, (**a**) card association network; (**b**) discovering new group; (**c**) expansion of merchants.

After the spread of seed cards based on the card-to-card association network, this experiment identifies more groups. In the case of only using the seed cards, the model can only find 344 groups, whereas when suspicious cards are added the model can find 414 groups. Figure 6a is a sub-graph of the card association network. The red represents the seed cards selected by the rule, and the yellow is the suspicious cards. Though suspicious cards are similar in behavior tp the seed cards, they cannot comply with the fixed parameter of the expert rules. Thus suspicious cards are easily omitted by the rules and are identified as normal cards.

- Discover a new group

Considering that the occasional transaction of the seed card leads to the incorrect association of normal merchant nodes, the connection between merchants with weak correlations will be omitted when constructing a merchant network. Without using local graph clustering, the number of seed cards involved in all merchants of group A is 19. Due to the weak connection, these merchants are not treated as groups. After adding the local graph clustering, the cash-out suspicious cards expand from 19 to 45, which strengthens the connection between the group merchants which are recognized as groups, as shown in Figure 6b.

- Expansion of merchants in existing groups

Group B has a total of 18 merchants after the spread of the seed cards. In this group, the suspicious cards missed by the rules are similar to seed cards. The number of merchants expands from 6 to 18, and the overall business effect has been greatly improved, as shown in Figure 6c and Table 5.

#### 3.3.2. Precision and Hit Rate

In our experiment, the merchant precision reaches 86% after confirmation and the model covers 145 merchants from 195 known risky merchants in the dataset, which shows that this method can mostly identify cash-out groups (Table 6).

In addition, 178 other group merchants in four groups are firstly discovered and verified as cash-out merchants belonging to the same acquirers with 195 known risky merchants. Compared with traditional financial rules and a supervised learning algorithm, this model can more comprehensively identify groups of cash-out merchants. Comparing the expert rule, GBDT algorithm and this model, this model is superior in terms of both precision and in group recognition (Table 7). The existing experienced expert rules are provided by the worldwide payment processor. The GBDT algorithm with confirmed cash-out groups as negative samples is used.

#### 3.3.3. Types of Merchant Network

**Figure 7:**
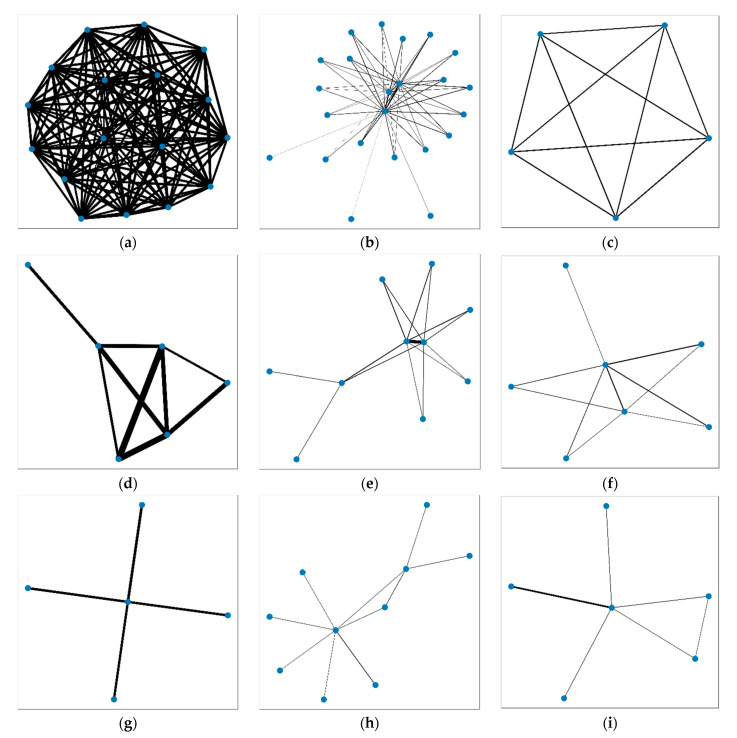
Nine types of merchant network rank: (**a**) AA; (**b**) AB; (**c**) AC; (**d**) BA; (**e**) BB; (**f**) BC; (**g**) CA; (**h**) CB; (**i**) CC.

For each rank type, typical groups are selected for analysis and to display, and the results are shown in Figure 7. Figure 7a shows a merchant group ranking AA, with the closest nodes and the most density. Figure 7b shows a merchant group ranking AB, with the most closeness between nodes but not very high density. Figure 7c shows a merchant group ranking AC, with a single node having one edge with the other. Figure 7d shows a merchant group ranking BA, with very close and dense relations between partial sections, but not the whole nodes. Figure 7e shows a merchant group ranking BB, with good closeness but common shared edges, and the *scale* value in Equation (6) is low although multiple edges exist in the subgraph. Figure 7f shows a merchant group ranking BC, with a high *scale* value in Equation (6) which cannot be seen in the subgraph. Finally, Figure 7g–i correspond to a merchant group ranking CA, CB and CC.

#### 3.3.4. Group Evolution Analysis

As time passes, the characteristics of the group’s cash-out behavior will also change. The life cycle of some groups may only exist for a few months, while some groups may always have illegal practices, and their scale may even continue to grow. Therefore, a time series analysis of the characteristics of the group portraits is carried out.

**Figure 8:**
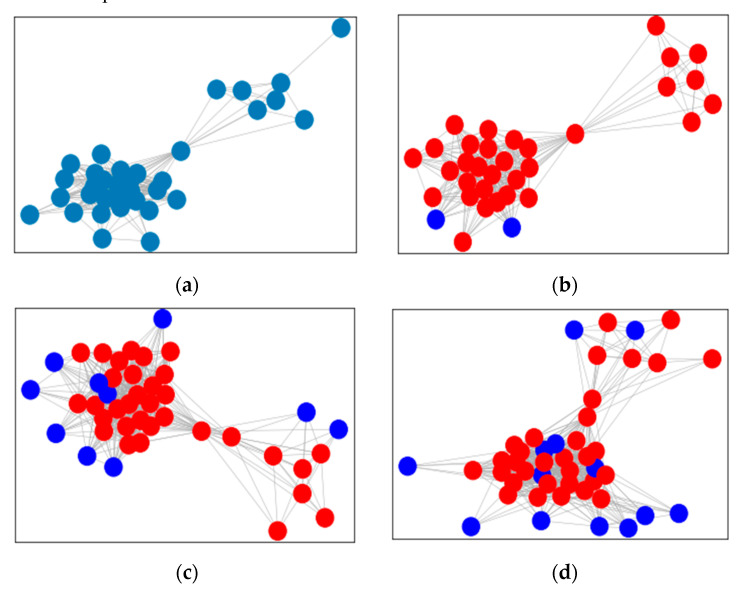
Change of group structure with time. (**a**) February; (**b**) March; (**c**) April; (**d**) May.

In Figure 8, a certain group structure has varied from February to April in 2020. The group has expanded and the blue nodes are new, while the red nodes are the same as the previous month.

## 4. Discussion

### 4.1. Rank Description

According to our rank calculation method, we ranked the 145 merchants recognized by the model in the dataset (Table 8). It can be seen that nearly half of the merchants belong to the group of AA rank, and no merchant is in the group of CC, which further verifies the rationality of the group classification method.

### 4.2. Consideration of Future Research

In the future, the graph network, including suspicious card diffusion and weight of the edge between merchant nodes, could be improved. In addition, graph neural networks (GNN) can be introduced for embedded representation learning of merchant nodes in the network. In terms of the confirmation of more group labels, the group classification and grading rank method can be optimized.

## 5. Conclusions

We present a model scheme for the mining of cash-out groups. The model proposes to use the technology of the associated network to link the originally independent merchants to build the associated network between merchants, so as to express the close relationship between the merchants more clearly. We use the graph embedding technology for each merchant learning to obtain a unique embedded representation, thereby mapping the merchant structure in the non-Euclidean space to the Euclidean space. In the vector space, clustering algorithms are used to locate each merchant in a specific community. In the network, the more closely connected the merchants are, the more similar the embedded representation. On this basis, the framework of this model portrays the characteristics of the group according to the transaction behavior, and enriches the characteristics of different groups. The IoT information is fully made use of.

On a certain 6-month real transaction dataset, the model accurately captures a major known case and covers 145 merchants from 195 known risky merchants in the dataset. In addition, it identifies four new groups, including 178 merchants, showing significant effects.

## Figures and Tables

**Table 1:** List of terminology.

Terminology	Description
Cards	Payment tool in the form of physical cards or virtual card number stored in mobile devices, like payment card tokenization
Seed cards	A collection of cards captured by expert rules
Suspicious cards	A collection of cards obtained after the spread of seed cards, including seed cards
Shared cards	A collection of cards that have transactions between merchants
Merchant acquiring by bank	Merchants with the acquirer which is a bank
Merchant acquiring by non-bank	Merchants with the acquirer which is a third party payment institution

**Table 2:** Comparison of three clustering algorithms.

	k-Means	Mean_Shift	DBSCAN
Whether specifies the number of clusters	√	×	×
Whether to be initialized	√	×	×
Whether sensitive to parameters	√	√	√
Whether handles non-convex clusters	×	√	√

**Table 3:** The entire message format.

Number	Data Element	Description
1	Transaction mode	Online/face-to-face
2	Card currency class	Single/dual currency
3	Acquirer number	
4	Issuer number	
5	PAN hash value	Primary Account Number
6	Date	YYYY/MM/DD
7	Time	
8	Transaction code	Purchase/authorization completion
9	Transaction channel	ATM/PoSComputer/mobile device, etc.
10	Interaction mode	Magnetic stripe/chip/card not present
11	Transaction amount	
12	Merchant type	Department store/hotel/restaurant, etc.
13	Merchant name	
14	Merchant number	
15	Money settlement class	T+0/T+1, etc.
16	Merchant info as device in IoT	IP address, device name, etc.
17	Phone/PC info as device in IoT	IP address, SEID, etc.
18	Response code	

**Table 4:** Seed cards in typical cash-out modes.

	Large Amount Mode	Periodic Mode	Abnormal Mode	Balance Mode	Equal Amount Mode
Number of seed cards	105,406	1,032,691	79,773	31,554	120,295

**Table 5:** Comparison of three clustering algorithms.

	Group B (Just Seed Cards)	Group B (with Suspicious Cards)
Number of merchants	6	18
Number of cards	123	323
Transaction amount (CNY million)	2.5	14

**Table 6:** Model effect of this model.

Number of Merchants in the Groups by Model	21,695
Number of merchants in the groups after confirmation	18,658
Merchant precision	86%
Number of merchants in a known case	195
Number of merchants in a known case recognized by model	145
the case hit rate	74.4%

**Table 7:** Comparison of model effect.

	Expert Rule	GBDT	This Model
Merchant precision	62.3%	78.4%	86%
Group recognition	None	None	Hit rate 74.4%, and 4 groups newly revealed including 178 merchants *

* There are 30,586 merchants totally acquiring using four banks as acquirers.

**Table 8:** The rank distribution of the groups hit by the model.

Rank	Number of Groups	Percentage of Groups	Number of Merchants	Proportion of Merchants
AA	4	28.6%	72	49.7%
BA	1	7.1%	38	26.2%
BB	6	42.9%	20	13.8%
BC	1	7.1%	1	2.2%
CB	2	14.3%	14	9.7%
CC	0	0	0	0
Total	14	100%	145	100%
